# Pain Following Stroke: A Population-Based Follow-Up Study

**DOI:** 10.1371/journal.pone.0027607

**Published:** 2011-11-15

**Authors:** Henriette Klit, Nanna B. Finnerup, Kim Overvad, Grethe Andersen, Troels S. Jensen

**Affiliations:** 1 Danish Pain Research Center, Aarhus University Hospital, Aarhus, Denmark; 2 Department of Epidemiology, Aarhus University, Aarhus, Denmark; 3 Department of Neurology, Aarhus University Hospital, Denmark; Innsbruck Medical University, Austria

## Abstract

**Background and Purpose:**

Chronic pain is increasingly recognized as a consequence of stroke. This study aimed to describe the prevalence and pain types of new onset chronic pain (“novel pain”) in patients with stroke compared with a randomly selected reference group from the general population and to identify factors associated with pain development in stroke patients.

**Methods:**

In a population-based follow-up design, development of chronic pain after stroke was assessed by a questionnaire sent to consecutive stroke patients, registered in a Danish national stroke database, two years after their stroke. A randomly selected sex- and age-matched reference group from the same catchment area received a similar questionnaire about development of new types of chronic pain in the same time period. A total of 608 stroke patients and 519 reference subjects were included in the study.

**Results:**

Development of novel pain was reported by 39.0% of stroke patients and 28.9% of reference subjects (OR 1.57, CI 1.21-2.04), and was associated with low age and depression in a multivariate model. Daily intake of pain medication for novel pain was reported by 15.3% and 9.4% of the stroke and reference population, respectively. Novel headache, shoulder pain, pain from increased muscle stiffness, and other types of novel pain were more common in stroke patients, whereas joint pain was equally common in the two groups.

**Conclusions:**

Development of chronic pain is more common in stroke patients compared with sex- and age-matched reference subjects. Evaluation of post-stroke pain should be part of stroke follow-up.

## Introduction

Stroke is the third leading cause of mortality and the major cause of long-term disabilities, such as hemiparesis, language problems and cognitive deficits, in the developed world [1] [2]. The reported prevalence of chronic pain in stroke survivors varies considerably with figures ranging from 11 to 53% [3]–[13]. This variability may be related to differences in criteria and methodologies used. Only few of these studies are population based and none of them have included a reference group.

Post stroke pain (PSP) is often considered to be identical to central post stroke pain (CPSP) also known as thalamic pain (e.g. [14]). However, CPSP is a specific neuropathic pain condition in which pain is due to a lesion of the somatosensory pathways within the central nervous system, i.e., those pathways that conduct information of noxious and non-noxious stimuli from the periphery to the brain. The stroke lesion causing CPSP may be located either within or outside the thalamus [15]. It is now clear that patients with stroke may suffer from a range of other pain types such as hemiplegic shoulder pain [16], [17], musculoskeletal pain [4], [7], [18], and headache [7], [11], [12], besides central post-stroke pain (CPSP) [19]–[21]. Patients may have several types of PSP concomitantly [3], [11], and often have a history of chronic pain prior to their stroke [7], [22]. A detailed and a priori delineation of these types of pain and how they each influence the quality of life in this group of patients has not been systematically done before.

We combined clinical data from a national database of consecutive patients admitted with a stroke with data from a postal questionnaire mailed 2 years after stroke. The aim of the study was 1) to describe the prevalence and pain types of new onset chronic pain (“novel pain”) in stroke patients compared with a randomly selected sex- and age-matched reference group from the general population and 2) to identify factors associated with pain development in stroke patients. Part of this study has been reported elsewhere [23] and identified CPSP in this stroke sample has been published recently [13].

## Materials and Methods

### The NIP database

All hospitalized acute stroke patients in Denmark are diagnosed according to the WHO criteria (ICD-10) and registered in a National Indicator Project database (NIP) [24]. Patients with intracerebral hemorrhage (I 61), cerebral infarction (I 63), and unspecified stroke (I 64) were included in the study, while patients with transient ischemic attacks (G 45) or subarachnoid hemorrhages (I 60) were excluded. The NIP stroke database contains information on stroke risk factors, severity, evaluation, and treatment, and is subject to regular systematic audits. Stroke severity is measured on admission using the Scandinavian Stroke Scale (SSS), a validated 9-item scale with scores between 0 and 58, where a high score indicates preserved function [25]. Disability and activities of daily living (ADL) are measured on day 7 (±2 days) after stroke using the Barthel Index (BI) [26]. Hospital files were not available for this study.

### Study Design

All patients hospitalized with a stroke diagnosis in the County of Aarhus (population 657,671 inhabitants, January 2005) between March 1, 2004 and February 28, 2005 [27] were included in the study. A questionnaire about the development of novel pain after stroke onset was sent out in October 2006 to all 964 surviving Danish patients (median days from stroke 794.5 (range 588–1099)). A similar questionnaire about the development of novel chronic pain within the last two years was sent to 957 (F = 456, M = 501) sex– and age-matched reference subjects. The reference group was randomly selected from the Danish general population in the same catchment area and was identified through the Central Office of Civil Registration. A reminder was sent out to non-responders after one month. If needed, participants were contacted by telephone for clarifying responses (152 stroke patients and 49 reference subjects). Proxy responders were allowed if the subjects could clearly communicate their pain experience.

### Questionnaire: General

The questionnaire included sections on demographics, medical and stroke history, increased muscle tone and spasms, sensory symptoms, pain, and concomitant diseases (see the English translation of the questionnaires in the supplementary material, Figure S1, S2). In addition, all subjects were asked to draw areas of abnormal sensitivity on a body chart and to rate their quality of life and health on a rating scale from 0 to 10 (0 = “bad” and 10 = “excellent”).

### Questionnaire: Pain

New onset chronic pain (in the following called “novel pain”) was defined as constant or remitting pain lasting more than 3 months and with onset at or after the stroke in patients and within the last 2 years in reference subjects. Subjects reporting development of novel pain were asked specifically about pain due to increased muscle stiffness, headache, shoulder pain, other joint pain, or “other pain” and to fill out a section on pain interference, including questions on how the pain affected their sleep, quality of life, mood, social life, and activities of daily living. Each pain interference item was rated on a 5 point scale: not at all, a little, some, quite a lot, and very much.

Subjects indicating development of “other pain” were asked to indicate the area of pain on a body chart; to score the intensity of their worst pain within the last week on a numeric rating scale (NRS) from 0–10, where 0 equals “no pain” and 10 “worst possible pain”; and to answer questions about the pain quality (from S-LANSS) [28]. A subset of patients who had indicated the presence of “other pain” and were suspected of central post-stroke pain (n = 51), were invited for a clinical examination (data reported elsewhere) [13].

### Ethics statement

The study was approved by the local ethical committee (the Central Denmark Region Committees on Biomedical Research Ethics; ID 20060083), the steering group of the NIP database, and the Danish Data Protection Agency (ID 2006-41-6779) and was performed according to the Declaration of Helsinki. An accompanying letter was sent out with the questionnaire. Only patients who gave written consent to further contact were solicited by telephone. This procedure was approved by the ethical committee.

### Statistics

Only subjects who had completed the screening question on novel pain (“Have you developed chronic pain following or in connection with the stroke, e.g., headache, joint pain or other pain in the body or the face?”) were included. Pain frequencies are based on the total number of included responders unless otherwise stated; responders with missing information to an item were excluded from the specific analysis.

Age was divided into 3 strata (<65, 65–74, and ≥75 years). Statistical analysis was performed using Intercooled Stata version 9.1 software (StataCorp LP, College Station, Texas, USA). Data are presented as mean and standard deviation (SD), with 95% confidence intervals (CI) or as median with 10% and 90% percentiles (p10-90) or range. P-values less than 0.05 were considered statistically significant. Parametric data were analyzed using Student's t-test. Non-parametric data were analyzed using Mann-Whitney and Kruskal-Wallis (rank sum). Dichotomous data were analyzed using Pearson's chi-square test and Fisher's exact test. Odds ratios are presented with 10% and 90% CI. A logistic regression analysis was performed to clarify the impact of variables identified to be associated with development of novel pain in the whole cohort and in stroke patients separately.

## Results

### Demographics

**Figure 1:**
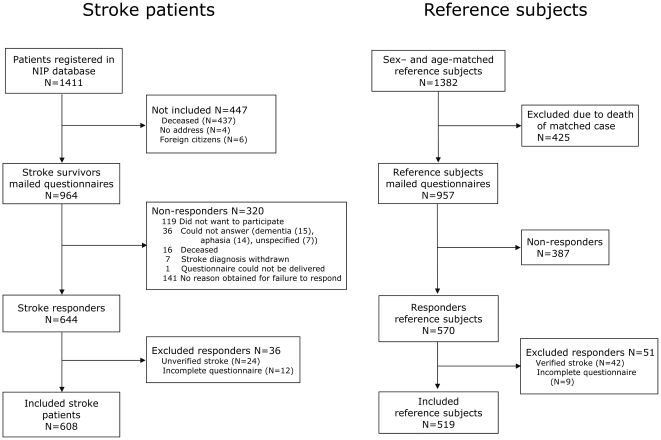
Study flowchart. Study flowchart of stroke patients (left side) and reference subjects (right side).

A total of 1411 patients were registered with stroke in the database. Of the 964 surviving stroke patients, 644 returned the questionnaires (response rate of 66.8%); 550 returned the primary questionnaire and 94 the reminder (figure 1). A total of 36 stroke responders were excluded: 24 denied having had the stroke episode and 12 had not completed the pain section of the questionnaire, leaving 608 (F = 268, M = 340) included stroke subjects (63.1%). Patient characteristics are seen in Table 1 and Table S1. Included stroke patients were younger and less severely affected by the stroke than the non-included.

**Table 1:** Comparison between included stroke patients and reference subjects based on data from the questionnaires.

	Included subjects	
	Stroke patientsN = 608	Reference subjectsN = 519
Included responders		
Primary questionnaire	520	471
Reminder	88	48
Median age (years)	72.6	71.0
Median age females (years)	74.9	74.0
Median age males (years)	70.9	69.0
Male percentage (%)	55.9	58.4
Days stroke to questionnaire, median (range)	794.5 (588–1099)	-
SSS score, median (p10-90)	50 (26–58)	-
Overall QoL (NRS), median (p10-90)¤	7 (3–9)	8 (5–10)
Overall QoH (NRS), median (p10-90)#	6 (3–9)	8 (5–10)
Self-reported diabetes (%)	14.1	7.3
Self-reported depression (%)	20.9	5.6
Self-reported CVD (%)	33.4	16.6
Cohabiting/married (%)	58.6	64.7
Living in nursing home (%)	12.7	1.9
Novel pain (%)	39.0	28.9
Headache (%)	10.5	2.3
Shoulder pain (%)	15.1	9.8
Other joint pain (%)	22.0	18.5
Pain due to muscle stiffness and spasms (%)	17.4	5.2
Other pain (%)	22.9	13.5

A total of 570 out of 957 reference subjects returned the questionnaire (59.6%): 517 returned the primary questionnaire and 53 the reminder. Of the responders, 51 were excluded due to either stroke (n = 42) or an incomplete pain section (n = 9), leaving 519 (F = 216, M = 303) included reference subjects (54.2%) (figure 1).

The median response proportion to each question was 94.7% (range 82.2–100%). The lowest response proportion was observed in the questions describing the impact of pain on social life.

Stroke patients had a higher response proportion than reference subjects. There was no statistically significant difference in gender distribution and age between stroke patients and reference subjects (table 1). Female subjects in both groups were significantly older than male subjects. Stroke patients had a higher reported frequency of diabetes and depression than reference subjects, whereas joint disease, gastrointestinal problems, and other pain-causing diseases were equally common.

### Incidence of novel pain in stroke patients compared with reference subjects

**Figure 2:**
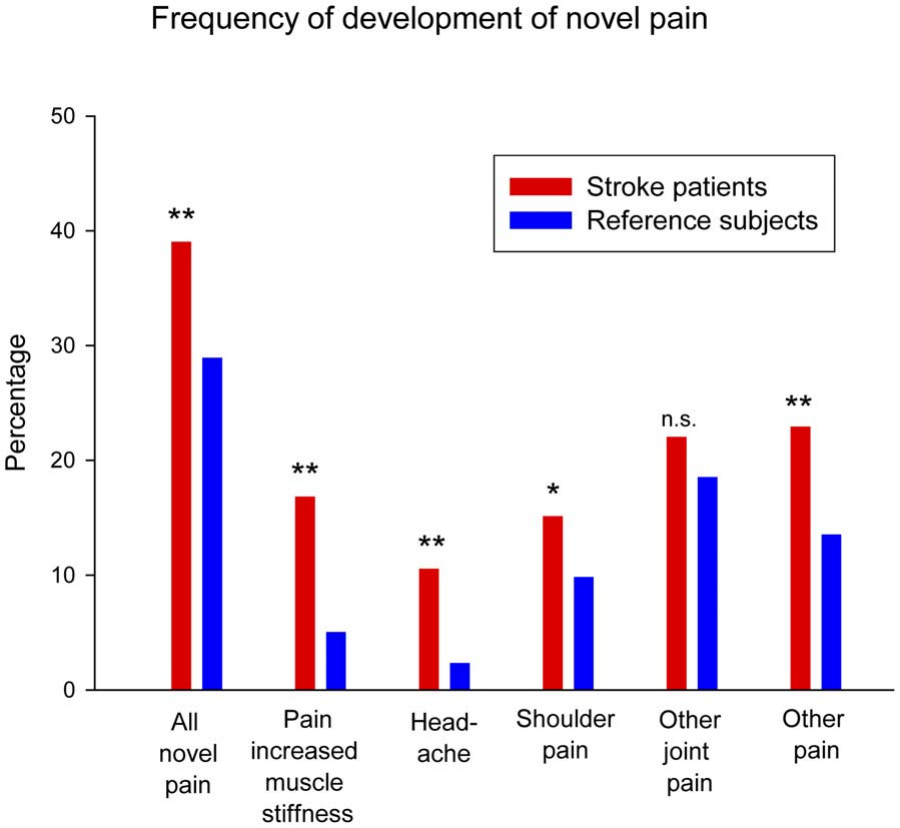
Frequency of development of novel pain in stroke patients and reference subjects. The reported prevalence of novel pain development in stroke patients (red columns) and reference subjects (blue columns). Stroke patients had a significantly higher prevalence of pain due to muscle stiffness or spasms, headache, shoulder pain, and other pain. * *p*<0.05, **p<0.001.n.s. not significant.

Development of novel pain after stroke or within the last 2 years was reported by 39.0% (35.1–43.0%) of stroke patients compared with 28.9% (25.0–33.0%) of reference subjects, OR 1.57 (1.21–2.04) (Figure 2). The highest frequency of novel pain was in the youngest age group of stroke patients in contrast to the reference group, where the pain frequency increased with age (Table S2).

In a univariate model, stroke was associated with a higher odds ratio (1.57) for development of novel pain as compared to the reference group (Table 2, model 1). This association was only slightly lower (OR 1.53) when correcting for age, gender, and diabetes in a multiple regression analysis (Table 2, model 2). When depression was included in the analysis (Table 2, model 3), there was no longer a significant difference in odds between stroke patients and reference subjects (OR 1.28, CI: 0.98–1.66). We tested for possible effect modification of gender, age, diabetes, and depression and found that a likelihood ratio test after estimation was significant for age (p = 0.0082), but not for gender (p = 0.25), diabetes (p = 0.49), and depression (p = 0.076). Self-reported cardiovascular disease (CVD) was not included in the analysis because the self-reported data to this item also covered ischemic pain in lower limbs.

**Table 2:** Logistic regression.

Model	Odds ratio for pain in stroke patients vs reference subjects	Comment
**Univariable logistic regression**		
Model 1	1.57 (CI 1.22–2.02)	Odds ratio for pain development in stroke patients as compared to reference subjects
**Multiple variable logistic regression**		
Model 2	1.53 (CI 1.19–1.97)	Confounder analysis including self-reported diabetes, gender, agegroup
Model 3	1.28 (CI: 0.98–1.66)	Confounder analysis including self-reported diabetes, gender, agegroup and **depression**
**Stratified analysis (Model 2)**		
Gender	1.76 (1.24–2.49)	Males
	1.29 (0.89–1.86)	Females
Age	2.67 (CI: 1.69–4.22)	Age<65 years
	1.33 (CI: 0.81–2.18)	Age 65–74 years
	1.09 (CI: 0.74–1.61)	Age>75 years
Diabetes	1.20 (CI: 0.54–2.67)	With diabetes
	1.57 (CI: 1.20–2.06)	Without diabetes
Depression	2.82 (CI: 1.22–6.52)	With depression
	1.17 (CI: 0.88–1.54)	Without depression

Odds for pain development in stroke patients vs. reference subjects.

Daily intake of pain medication was required by two-thirds of the subjects with development of novel pain, corresponding to the use of daily pain medication for novel pain in 15.3% of the stroke population and 9.4% of the reference population (p = 0.003). Pain interference was higher in stroke patients compared with reference subjects with respect to quality of life (p = 0.006), mood (p = 0.003), social life (p<0.001), and activities of daily living (p<0.001), but not with respect to interference of sleep (p = 0.69) (mean response rate 83.5% (81.9–85.3%)). In the subjects reporting development of novel pain, there was a significant association between high pain interference and depression in stroke subjects (p<0.001), but not in control subjects (p = 0.15).

### Subtypes of novel pain

#### Headache

Development of headache was more common in stroke patients than in reference subjects (10.5% vs. 2.3%, p<0.001), OR 4.97 (2.62–10.23) (Figure 2, Table 2).The frequency and severity of the headache were the same in the two groups. In stroke patients with novel headache, 63.5% reported headache more than 7 days per month and 46.9% had severe or unbearable headache. A history of headache prior to the development of the novel headache was common. The proportion of novel headache was highest in the youngest stroke patients (p = 0.03), but equally common in men and women.

#### Shoulder and other joint pain

Development of shoulder pain was more common in stroke patients than in reference subjects (15.1% vs. 9.8%, p<0.001), OR 1.64 (1.12–2.40) (Table S2). In stroke subjects, the proportion of novel shoulder pain decreased with age (p = 0.02).

Development of pain from other joints was equally common in stroke patients and reference subjects (22.0% vs. 18.5%, p = 0.14), OR 1.25 (0.92–1.69), but more common in women compared with men (p = 0.002). Novel pain from multiple other joints was common in stroke patients and included pain in hips, ankles, feet, knees, neck, back, wrist, fingers and elbows.

#### Muscle stiffness, spasms and pain

Muscle stiffness or spasms were reported by 32.8% of stroke subjects compared with 9.8% of reference subjects (p<0.001), OR 4.50 (3.16–6.46). Pain directly due to muscle stiffness or spasms was reported by more than half of the subjects with these symptoms, corresponding to 17.4% of stroke patients and 5.2% of reference subjects (p<0.001), OR 3.82 (2.41–6.28) (Table S2).

#### Other novel pain

In stroke patients, 22.9% had developed other pains (i.e., not pain from increased muscle stiffness or spasms, headache, shoulder, or other joints) compared with 13.5% of reference subjects (p<0.001), OR 1.90 (1.37–2.64) (Table S2). In stroke patients, the proportion of patients reporting other novel pain tended to decrease with age (p = 0.08). Diabetes was not associated with development of other novel pain.

The localization of other pain is summarized in Figure S3. The area of other novel pain (“worst pain”) was more often unilateral in stroke patients compared with reference subjects (Table S1). A sensation of “pins and needles” and unpleasantness or pain in response to light touch was more common in stroke patients than reference subjects reporting other novel pain, whereas burning pain was described equally common in the two groups (Table S1).

#### Concomitant types of novel pain

Stroke patients were more likely to report development of more than one concomitant pain type compared with controls (61.2% vs. 48.0%, p = 0.011). The likelihood of reporting several novel pain types concomitantly decreased significantly with age in stroke patients (p<0.001), but not in reference subjects (p = 0.40).

### Factors related to pain in the stroke patients

The stroke diagnosis did not differ between stroke patients with or without development of novel pain (p = 0.32), but the median SSS score on admission was lower, i.e. indicating a more severe stroke, in stroke patients reporting novel pain than in patients without novel pain (p = 0.0018) (Table 3). A history of prior stroke was more common in stroke patients with novel pain compared with stroke patients without novel pain (27.9% vs. 20.1%, p = 0.027), whereas a diagnosis of atrial fibrillation was more common in stroke patients without novel pain (p = 0.031). There were no significant differences between the stroke patients with and without novel pain with respect to other risk factors at the time of stroke.

**Table 3:** Comparison between stroke patients and reference subjects with development of novel types of pain vs. no pain development at time of questionnaire.

	Stroke patients		
	Pain	No pain	P-value
Included responders	237	371	-
Primary questionnaire	193	327	**0.022**
Reminder	44	44	
Percentage of included responders, % (95% CI)	39.0 (35.1–43.0)	61.0	-
Median age (years)	70.8	73.2	**0.018**
Median age females (years)	72.4	77.0	**0.0075**
Median age males (years)	68.9	71.2	0.28
Male percentage (%)	52.3	58.2	0.15
Days stroke to questionnaire, median	801	794	0.98
SSS score, median (p10-90)	48 (24–58)	51 (30–58)	**0.0018**
Overall QoL (NRS), median (p10-90)¤	5 (2–8)	7 (4–10)	**<0.001**
Overall QoH (NRS), median (p10-90)#	5 (2–8)	7 (4–10)	**<0.001**
Self-reported diabetes (%)	15.6	13.2	0.41
Self-reported depression (%)	34.6	12.1	**<0.001**
Self-reported CVD (%)	45.2	25.9	**<0.001**
Cohabiting/married (%)	57.0	59.6	0.53
Living in nursing home (%)	13.9	11.9	0.46

In a multiple logistic regression of all included stroke patients (n = 608) (Table 4), low age, and depression were identified as significant risk factors for development of post-stroke pain. When the SSS score was included in the same analysis (n = 527), a low SSS score was also a significant risk factor for pain development.

**Table 4:** Logistic regression in stroke patients only.

Variable	Odds ratio for development of pain after stroke (95% CI) (n = 608)#	P-value	Odds ratio for development of pain after stroke (95% CI) (n = 527)¤	P-value
Diabetes (vs no diabetes)	1.08 (0.65–1.78)	0.77	1.08 (0.63–1.88)	0.76
Males (vs females)	0.77 (0.53–1.11)	0.16	0.90 (0.61–1.32)	0.58
Depression (vs no depression)	3.43 (2.25–5.25)	**<0.001**	**3.13 (1.99–4.91)**	**<0.001**
Diagnosis (vs hemorrhage)			NA	-
Infarction	0.73 (0.43–1.26)	0.27		
Unspecified	1.09 (0.57–2.09)	0.79		
Age (vs <65 years)				
Age 65–74 years	0.57 (0.36–0.90)	**0.015**	0.57 (0.35–0.93)	**0.026**
Age≥75 years	0.65 (0.43–0.99)	**0.043**	0.63 (0.40–0.98)	**0.041**
SSS (<45)	NA	-	0.60 (0.40–0.89)	**0.011**

# Multiple regression model of all included stroke patients, including the same variables as in the multiple variable resgression analysis of all included subjects (table 2).

¤ Multiple variable regression model including the SSS ( = Scandinavian Stroke Score).

## Discussion

To our knowledge, this is the first published population-based study on PSP including a control group. Stroke patients compared with age and sex-matched reference subjects more often reported development of novel pain including pain due to increased muscle stiffness, headache, shoulder pain, and other types of novel pain. Two-thirds of the patients with development of novel pain were taking daily pain medication, corresponding to 15% of the total stroke population. Taken together these findings indicate that novel pain after stroke is a symptom with a major impact on the stroke patient, in addition to the other well-known motor and cognitive sequelae that often accompany stroke. Former studies have shown that stroke represents the disease condition associated with the highest degree of disability [2]. The present findings raise the possibility that pain is another contributing factor to the disability seen in stroke survivors.

In an univariate model in this study, stroke patients were more likely to report development of chronic pain following stroke (39.0%) compared with reference subjects (28.9%), developing pain within the last 2 years (OR: 1.57). The difference between the two groups (risk difference 10.1%, CI 4.6–15.6%) can be interpreted as the proportion of pain that is stroke related, and supports findings from other studies [5], [7], [8]. Consistent with previous observations pain was associated with depression.

When correcting for possible confounders in a multiple regression analysis that included depression, there was a significant reduction in the difference in odds between stroke patients and reference subjects. These findings suggest that the higher odds for pain development in stroke patients were partly due to an associated depression, rather than stroke *per se*. The difficulty in dissecting the relationship between depression and pain in general and pain due to stroke in particular is underscored by the fact that a) risk of depression is increased after stroke [29], b) patients with chronic pain are more likely to report depression [30], and c) depressed patients are more likely to have pain than non-depressed patients [31]. In the present study, we found a significant association between high pain interference on mood and depression in stroke patients reporting development of pain, but not in control subjects reporting pain. Clinical and experimental studies have shown a high concordance of depression and pain. It is still unclear if chronic pain and mood disorders share common pathophysiological mechanisms or whether they are both caused by separable and distinct mechanisms. It is of interest to speculate on the mechanisms behind this higher frequency of depression in stroke patients compared with reference subjects. The mechanisms underlying pain and depression have been linked to disturbances in the monoaminergic neurotransmission systems originating in the brainstem and projecting down both into the spinal cord and into the forebrain [30], [32], [33]. Disruption of serotonergic and noradrenergic systems is likely to occur in patients suffering a stroke affecting the brainstem and subcortical structures [34], [35]. In chronic pain there is pharmacological evidence that restoring serotonergic and noradrenergic neurotransmission with specific serotonergic and noradrenergic re-uptake inhibitors can reduce pain in patients with peripheral or central neuropathic pain conditions (for review see Finnerup et al. 2010) [36]. Thus a more profound reduction of central serotonin and noradrenaline tone in stroke patients with pain than in the reference group with pain may be one possibility for the association of depression in the stroke pain group and not in the reference pain group.

In this study, and also in a recent study [12], pain was more prevalent in the youngest age group of stroke patients. This is in contrast to what is seen in the reference group, where the prevalence of novel pain, increased with age as we expected [37]. The mechanism for this higher pain frequency in the youngest stroke patients is not clear. It has been shown that stroke in the brainstem and thalamus more frequently are associated with central pain than other locations (for review see Klit et al [38]) and that posterior territory infarcts, including brainstem and thalamic strokes, are relatively more frequent in the younger age groups [39]–[41]. Whether this age-dependent effect may account for the present observation requires further studies.

The presence of specific subtypes of pain was assessed by asking about pain due to increased muscle stiffness including spasms, headache, shoulder pain, other joint pain, and “other pain”. The reported proportion of muscle stiffness or spasms was higher in stroke patients compared with reference subjects (OR: 4.50), and more than half of these subjects reported pain directly due to these symptoms. Our findings are in accordance with previous studies reporting a prevalence of spasticity of 17–38% using clinical assessment scales [42]–[44]. Development of chronic headache was more common in stroke patients than in reference subjects (OR: 4.97). In other studies, post-stroke headache has been reported by 10% [7], [12]. The present study supports the notion of headache being a common consequence of stroke, and we have previously suggested that the development of headache might be pathophysiologically linked to the stroke [45]. However, the high prevalence of post-stroke headache in this study may be partly ascribed to the routine use of dipyridamole in ischemic stroke patients at the time of data collection [46]. Development of shoulder pain was reported by 15% of stroke patients and 10% of reference subjects (OR: 1.64). In previous studies, the range of reported prevalence of post-stroke shoulder pain is wide, ranging from 6–64% [8], [16], [47], [48]. Differences in study populations and criteria used may explain this variability.

The strength of this study is the inclusion of a sex- and age-matched reference group randomly chosen in the same area as the stroke population. The inclusion of a control group is important, as chronic pain is common in all population groups, and in particular among the elderly [49], [50]. Stroke and reference subjects were comparable with respect to age, sex, and concomitant diseases apart from diabetes, CVD, and depression. To exactly determine the influence of stroke for the subsequent development of pain, the ideal control group would have the same risk factors as the study group.

The present study population was selected from a stroke database including 95% of all stroke patients in the area [51]. The NIP stroke database undergoes regular audit and the quality and reliability of the data are high. As regards the reliability of the information from the questionnaires, the overall quality of the responses was good and response rates of the individual questions were generally high (94.7%). In cases of uncertainty responders were contacted by telephone in order to clarify responses.

The present study has a few shortcomings. The response rate was not high, but is consistent with response rates of other questionnaire studies in stroke survivors [52], [53]. It is a retrospective study with a risk of recall bias. However, the reference group is assumed to be exposed to a similar bias, so it is unlikely that the retrospective character of the study would change the relative frequency of pain between the two groups. The pain prevalence before the study is not known and may therefore differ between the groups. The pain frequency increased from the primary questionnaire to the reminder in the stroke patients (37.1% vs. 50.0%, p = 0.022), but not in the reference group (29.0% vs. 27.1%, p = 0.77), implying that pain frequencies for stroke patients were not overrated in this study. Stroke severity has, in this and previous studies [7], [17], been associated to pain prevalence. In this study, the included stroke patients were less severely affected than non-responders; however, the study group is likely to be representative for the stroke survivors. The pain intensity was not recorded for all subgroups of pain but only for headache and novel types of pain. In these two latter types of pain, there was no difference in pain intensity between stroke patients and reference subjects. What is of importance in a study like this may not be the pain intensity per se, but whether the pain has an intensity that needs daily medication. In this study 15% of stroke patients with novel pain after stroke took daily medication for their pains compared with 9% in the reference pain group.

In conclusion, pain represents an important disability following stroke. In this population-based study, which included a sex and age-matched reference group, about 40% of the stroke patients had developed chronic pain within two years of their stroke and this pain was associated with depression and low age.

## Supporting Information

Figure S1 **English translation of the questionnaire to the stroke subjects.**
(DOCX)

Figure S2 **English translation of the questionnaire to the reference subjects.**
(DOC)

Figure S3 **Localization of other novel pain.** The reported location of worst “other pain” in stroke patients (red bars) and reference subjects (blue bars). A hemibody distribution of pain, i.e., pain localized to one side of the body, with or without involvement of the face and trunk, and pain in parts of both upper and lower limbs, was more common in stroke patients than in reference subjects (hemibody: 21.3% vs. 4.5%, p = 0.002; parts of upper and lower limbs: 22.1% vs. 4.6%, p = 0.002), whereas pain with other localizations, including wide spread pain, pain in multiple sites, back pain and neck pain, was more common in reference subjects (15.0% vs. 34.9%, p = 0.003).
(TIF)

Table S1 **Characterization of the included stroke patients at time of stroke based on NIP data.**
(DOC)

Table S2 **Characterization of stroke patients and reference subjects with development of all types of novel pain and subtypes of pain.**
(DOC)
