# Supplementary material for: Pain Following Stroke: A Population-Based Follow-Up Study
Source: PLoS One. 2011 Nov 15;6(11):e27607. doi: 10.1371/journal.pone.0027607 (PMC3216963; doi:10.1371/journal.pone.0027607)
Supplement: Figure S1 — English translation of the questionnaire to the stroke subjects. (DOCX) [file pone.0027607.s001.docx]

|  |  |  |  |
| --- | --- | --- | --- |

# *Chronic post-stroke pain*

# *- a questionnaire study*

If you need help to complete this questionnaire, please contact Dr. Henriette Klit at the Danish Pain Research Center, Aarhus University Hospital: tel. 8949 3287 (preferably between 9:00 and 13:00).

| **How to complete the questionnaire:**  *Please answer all questions and mark (with a cross) the answer that is most appropriate for you or fill in the missing information.* ***Example:***   \| A. Patient data \| \| \| \| \| \| --- \| --- \| --- \| --- \| --- \| \| Date of birth: \| \| Month: **March** \| Year: **1945** \| \| \| Gender: \| □ Male \| \| \|  \| \|  \| □ Female \| \| \|  \|   *You will be asked to indicate where you have your pain and sensory disturbances. Please hatch the areas on the figure that correspond to the areas of your body where you have pain or sensory disturbances.* ***Example:***   \| 2. Mark on the below figure where you have your pain. \| \| \| --- \| --- \| \| Example: *Pain in the right side of the face, right lower arm and low back pain.* \| 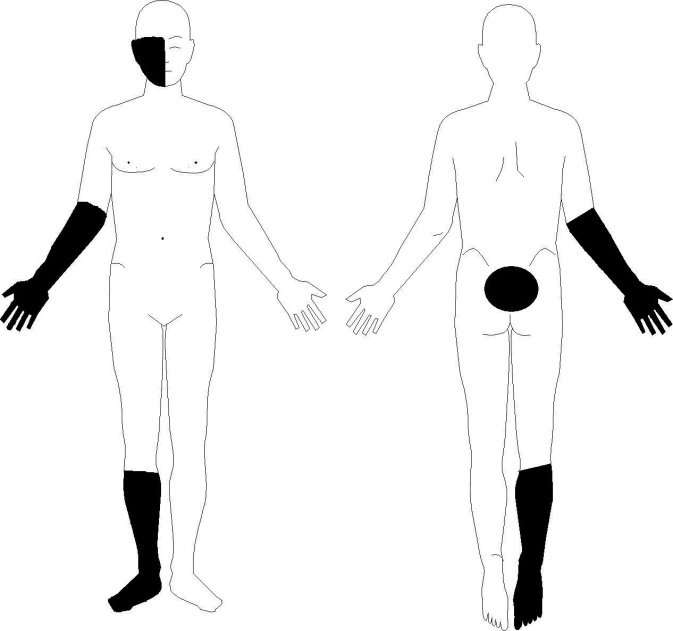 \| |
| --- | --- | --- | --- | --- | --- | --- | --- | --- | --- | --- | --- | --- | --- | --- | --- | --- | --- | --- | --- | --- | --- | --- | --- | --- |
| It is very important that you answer all questions of the questionnaire to the extent possible. |
| **Definition of words:**  **Chronic pain** means persistent or recurrent pain lasting for more than 3 months.  **Stroke** means a cerebrovascular accident, i.e. a brain thrombus or a brain hemorrhage. |

| A. Patient data | | | | | | | | | | | | | |
| --- | --- | --- | --- | --- | --- | --- | --- | --- | --- | --- | --- | --- | --- |
| 1. Date of birth: | Month:___________­­­__ | | | | | | Year:_______________ | | | | | |  |
| 2. Gender: □ Male | |  | | | |  | | | | | | |  |
| □ Female | |  | | | |  | | | | | | |  |
| 3. Marital status: □ Married/cohabiting | | | | | | | |  |  |  |  |  | |
| □ Living alone | | |  |  |  | | |  |  |  |  |  | |
| □ Other | | |  |  |  | | |  |  |  |  |  | |
| 4. Living facilities: □ Own home | | | | | | | |  |  |  |  |  | |
| □ Assisted living facility | | | | | | | |  |  |  |  |  | |
| □ Other | | |  |  |  | | |  |  |  |  |  | |

| B. Other diseases | | | | | | | | | | | | | | | |  |
| --- | --- | --- | --- | --- | --- | --- | --- | --- | --- | --- | --- | --- | --- | --- | --- | --- |
| 1. Do you have any of the following diseases or illnesses?  *Please mark the most appropriate answer for each line.* | | | | | | | | | | | | | | | |  |
|  | DISEASE | | | | | | | | NO | YES | | DO NOT KNOW | | | | |
| a. | Joint diseases  e.g. oosteoarthritis, arthritis, hip or knee surgery | | | | | | | |  |  | |  | | | | |
| b. | Other disease of the brain or nerves,  e.g. neuritis, herpes zoster, epilepsy | | | | | | | |  |  | |  | | | | |
| c. | Diabetes | | | | | | | |  |  | |  | | | | |
| d. | Gastrointestinal diseases | | | | | | | |  |  | |  | | | | |
| e. | Cardiovascular diseases  e.g. angina pectoris, leg pain on walking | | | | | | | |  |  | |  | | | | |
| f. | Depression | | | | | | | |  |  | |  | | | | |
| g. | Cancer | | | | | | | |  |  | |  | | | | |
| 2. Do you have other pain-related diseases? | | | | | | | | | | | | | | |  |  |
|  | □ YES Please specify__________________________________________ | | | | | | | | | | | | | |  |  |
|  | □ NO | |  | |  | | | | | | | | | |  |  |
| C. Stroke and headache | | | | | | | | | | | | | | |  |  |
| 1. Have you had a stroke (a thrombosis or hemorrhage)? | | | | | | | | | | | | | | |  |  |
|  | | □ YES If yes, go to the next question (2) | | | | | | | | | | | | |  |  |
|  | | □ NO **If no, please proceed to section D: Spasticity** | | | | | | | | | | | | | |  |
| 2. Have you had more than one stroke? | | | | | | | | | | | | | | |  |  |
|  | | □ YES | |  | | | | | | | | | | | |  |
|  | | □ NO | |  | | | | | | | | | | | |  |
| 3. Please indicate the date of your (last) stroke | | | | | | | | | | | | | | |  |  |
|  | | Month:__________ | | | |  | Year:______________________ | | | | | | | |  |  |
| 4. Have you previously, i.e. before the stroke, suffered from headache (e.g. tension-type headache or migraine)? | | | | | | | | | | | | | | | |  |
|  | | □ YES If yes, go to the next question (5) | | | | | | | | | | |  | | |  |
|  | | □ NO **If no, please proceed to section D: Spasticity** | | | | | | | | | | | | | |  |
| 5. Has your headache become more severe or frequent following or in connection with the stroke? | | | | | | | | | | | | | | | |  |
|  | | □ YES, it has become more severe | | | | | | | | |  | | |  | |  |
|  | | □ No change | | | | | | | | |  | | |  | |  |
|  | | □ NO, it has improved | | | | | |  | | |  | | |  | |  |

| D. Spasticity, muscle stiffness or spasms | | | |
| --- | --- | --- | --- |
| 1. Have you developed spasticity (increased muscle stiffness) or spasms (sudden, involuntary muscle contractions or movements) following the stroke? | | | |
|  | □ YES If yes, go to the next question (2) | |  |
|  | □ NO **If no, please proceed to section E: Sensory disturbances** | |  |
| 2. Do you experience pain due to spasticity or spasms? | | |  |
|  | □ YES |  |  |
|  | □ NO | |  |

| E. Sensory disturbances | | | | | | | | | |
| --- | --- | --- | --- | --- | --- | --- | --- | --- | --- |
| 1. Do you have sensory disturbances, i.e. decreased or changed sensory perception in an area of the body? | | | | | | | | | |
|  | □ YES If yes, please mark the area(s) on the below figure.   *Please see the example on page 2.* | | | | | | | | |
|  | □ NO **If no, please proceed to section F: Newly developed chronic pain** | | | | | | | | |
| *Please mark the areas of your sensory disturbances on the figure:* | | | | | 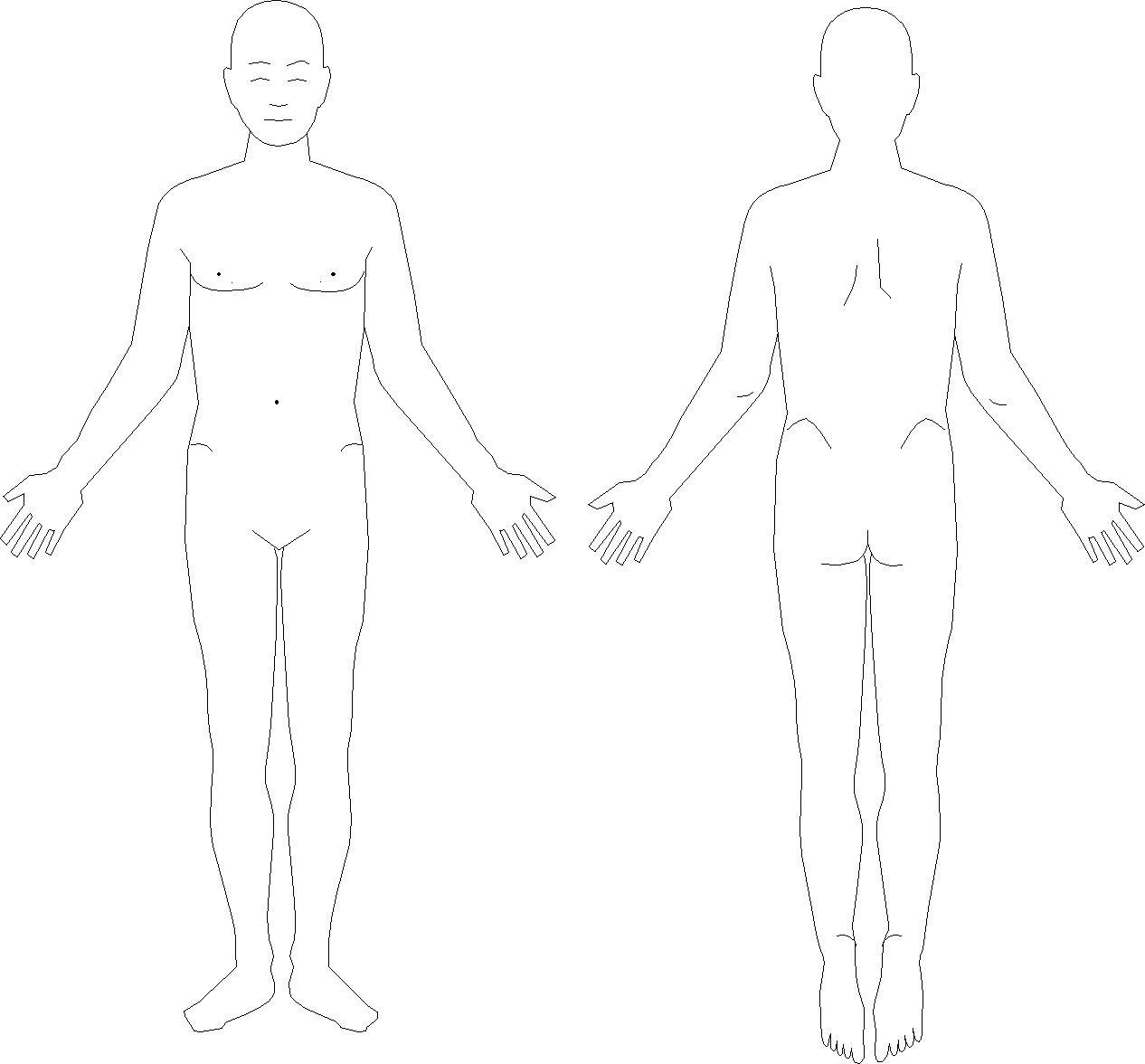 | | | | |
| 2. Have you developed unpleasant sensations or pain in the areas of sensory disturbances? | | | | | | | | | |
|  | □ YES | | |  | |  |  |  | |
|  | □ NO | | |  | |  |  |  | |
| 3. Have you developed hypersensitivity to touch or cold/heat in these areas? E.g. do you have pain or unpleasant sensations when washing in cold or warm water? | | | | | | | | | |
|  | | □ YES |  | | |  |  | |  |
|  | | □ NO |  | | |  |  | |  |

| F. Chronic pain developed following or in connection with your stroke | | |
| --- | --- | --- |
| 1. Have you developed chronic pain following or in connection with the stroke, e.g. headache, joint pain or other pain in the body or face? | | |
|  | □ YES If yes, go to the next question (section G: Headache) | |
|  | □ NO **If no, please proceed to section K: Final comments (last page)** | |
|  | |  |
| ***If you have not developed chronic pain following the stroke (e.g. headache, joint pain or other pain in the body or face) please proceed to section K: Final comments at the last page of the questionnaire.*** | | |
| ***If you have developed chronic pain following or in connection with the stroke, please go to the next question.*** | | |

| ***The next part of the questionnaire only concerns information on chronic pain developed following or in connection with the stroke.*** | | | |
| --- | --- | --- | --- |
|  | | |  |
| G. Headache | | | |
| 1. Have you developed chronic headache following or in connection with the stroke? | | | |
|  | □ YES If yes, go to the next question (2) |  | |
|  | □ NO **If no, please proceed to section H: Joint pain** |  | |

| 2. How often do you have a headache? | | | | | |  |
| --- | --- | --- | --- | --- | --- | --- |
|  | □ Less than one day per month | | |  | |  |
|  | □ 1-3 days per month | | |  | |  |
|  | □ 4-6 days per month | | |  | |  |
|  | □ More than 7 days per month | | |  | |  |
|  | □ Daily | | |  | |  |
| 3. What does your headache feel like when it is worst? | | | | | |  |
|  | □ Slight |  |  | |  |  |
|  | □ Moderate |  |  | |  |  |
|  | □ Severe |  |  | |  |  |
|  | □ Intolerable |  |  | |  |  |

| H. Joint pain | | |
| --- | --- | --- |
| 1. Have you developed chronic shoulder pain following the stroke? | | |
|  | □ YES |  |
|  | □ NO |  |
| 2. Have you developed other chronic joint pain following the stroke (e.g. in ankles, knees or hips)? | | |
|  | □ YES Location: _____________________________________________ | |
|  | □ NO | |

|  | I. Other newly developed chronic pain | | |  |
| --- | --- | --- | --- | --- |
|  | This section does not concern headache, pain due to spasticity or joint pain, but other newly developed chronic pain, e.g. pain and unpleasant sensations in arms or legs. | | |  |
|  | 1. Have you developed other chronic pain (persistent or recurrent pain lasting for more than 3 months) following the stroke? | | |  |
|  |  | □ YES If yes, go to the next question (2) | |  |
|  |  | □ NO **If no, please proceed to section J: Daily life with pain (page 10)** | |  |
| 2. Please mark on the below figure where you have your other pain, i.e. not headache, pain due to spasticity or joint pain, but other newly developed pain.  *Please see the example at page 2.* | | | | |
| *Mark the areas of your pain on the figure:* | | | 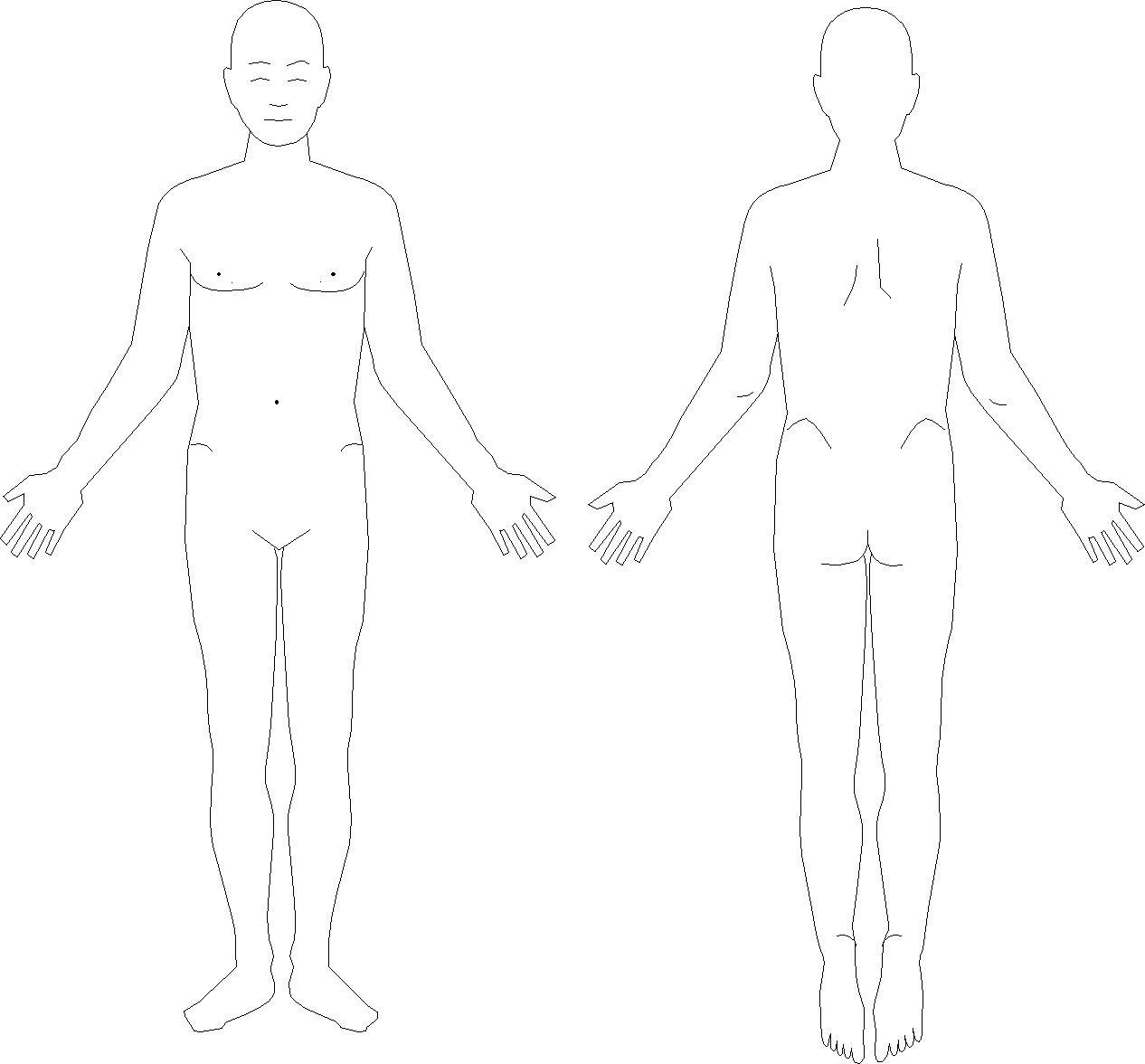 | |
| 3. Please indicate the location of your worst pain: ________________________________ | | | | |

| ***Questions 4-11* only concern your worst pain, i.e. the pain mentioned in Question 3.***  *Think about your pain in the last week and mark the most appropriate answers.* | | | | | | | | | | | | | | |
| --- | --- | --- | --- | --- | --- | --- | --- | --- | --- | --- | --- | --- | --- | --- |
|  | | | | | | | | | | | | | | |
| 4. On the scale below, please indicate how bad your pain (that you have shown on the above diagram) has been in the last week where: “0” means no pain and “10” means as severe as it could be.  *Please circle the number that best describes your pain.* | | | | | | | | | | | | | | |
| NO | | 0 | 1 | 2 | 3 | 4 | 5 | 6 | 7 | 8 | 9 | 10 | WORST POSSIBLE | |
|  |  | | | | | | | | | | | | |  |

| 5. In the area where you have pain, do you also have “pins and needles”, tingling or prickling sensations? | | |
| --- | --- | --- |
|  | □ NO | |
|  | □ YES | |
| 6. Does the painful area change color (perhaps looks mottled or more red) when the pain is particularly bad? | | |
|  | □ NO | |
|  | □ YES | |
| 7. Does your pain make the affected skin abnormally sensitive to touch? Getting unpleasant sensations of pain when lightly stroking the skin might describe this. | | |
|  | □ NO | |
|  | □ YES | |
| 8. Does your pain come on suddenly and in bursts for no apparent reason when you are completely still? Words like “electric shocks”, jumping and bursting might describe this. | | |
|  | □ NO | |
|  | □ YES | |
| 9. In the area where you have pain, does your skin feel unusually hot like burning pain? | | |
|  | □ NO | |
|  | □ YES | |
| 10. Gently rub the painful area with your index finger and then rub a non-painful area (for example, an area of skin further away or on the opposite side from the painful area). How does this rubbing feel in the painful area? | | |
|  | □ | I have the same sensation in both areas. |
|  | □ | I feel discomfort, like pins and needles, tingling or burning pain in the painful area that is different from the non-painful area. |

| 11. Gently press on the painful area with your finger tip and then gently press in the same way onto a non-painful area (the same non-painful area that you chose in the last question). How does this feel in the painful area? | | |
| --- | --- | --- |
|  | □ | I have the same sensation in both areas. |
|  | □ | I feel a numbness or tenderness in the painful area that is different from the non-painful area. |

**Modified from the S-LANSS by Bennett et al, The Journal of Pain, vol 6, No 3 (March), 2005:pp 149-158*

| J. Daily life with pain | | | | | | | | | | |
| --- | --- | --- | --- | --- | --- | --- | --- | --- | --- | --- |
| ***This section should be answered based on all your newly developed chronic pain, including headache, spasticity and joint pain.*** | | | | | | | | | | |
| 1. To what extent does your pain affect the following? *(one answer for each line)* | | | | | | | | | | |
|  | | NOT AT ALL | | A LITTLE | SOME | | A LOT | | A GREAT DEAL |  |
| Your sleep | |  | |  |  | |  | |  |  |
| Your quality of life | |  | |  |  | |  | |  |  |
| Your mood | |  | |  |  | |  | |  |  |
| Your social life | |  | |  |  | |  | |  |  |
| Daily activities (e.g. walking, bathing, household chores) | |  | |  |  | |  | |  |  |
| 2. Do you need medication for your pain? | | | | | | | | | | |
|  | □ YES If yes, go to the next question (3) | | | | | | |  | | |
|  | □ NO **If no, please proceed to section K: Final comments** | | | | | | | | | |
| 3. How often do you need to take medication for your pain? | | | | | | | | | | |
|  | □ Several times a day | |  | | |  | |  | | |
|  | □ Daily | |  | | |  | |  | | |
|  | □ Weekly | |  | | |  | |  | | |
|  | □ Less often | |  | | |  | |  | | |

| K. Final comments | | | | | | | | | | | | | | | |
| --- | --- | --- | --- | --- | --- | --- | --- | --- | --- | --- | --- | --- | --- | --- | --- |
| 1. How is your overall health?  *Please rate your overall health by circling the number that best describes your condition. ”0” means bad and ”10” means excellent.* | | | | | | | | | | | | | | | |
| BAD | | | 0 | 1 | 2 | | 3 | 4 | 5 | 6 | 7 | 8 | 9 | 10 | EXCELLENT |
|  |  | | | | | | | | | | | | | | |
| 2. How will you describe your overall quality of life?  *Please rate your overall quality of life by circling the number that best describes your quality of life. ”0” means bad and ”10” means excellent.* | | | | | | | | | | | | | | | |
| BAD | | | 0 | 1 | 2 | | 3 | 4 | 5 | 6 | 7 | 8 | 9 | 10 | EXCELLENT |
|  | | | | | | | | | | | | | | | |
| 3. May we contact you by phone or in writing for further clarification of your answers or in case we can offer you an in-depth examination? | | | | | | | | | | | | | | | |
|  | | □ YES **Your phone number**­­­­­­­­­­­­­­­_____________________________ | | | | | | | | | | | | | |
|  | | □ NO | | | |  | | | | | | | | | |

| ***You have now completed the questionnaire.***  ***Thank you very much!***  If you have further comments to this questionnaire, please write them here or contact us: tel. 8949 3287 (Henriette Klit, Danish Pain Research Center, Aarhus University Hospital) |
| --- |
