# Supplementary figures and images for: Pain Following Stroke: A Population-Based Follow-Up Study
Source: PLoS One. 2011 Nov 15;6(11):e27607. doi: 10.1371/journal.pone.0027607 (PMC3216963; doi:10.1371/journal.pone.0027607)

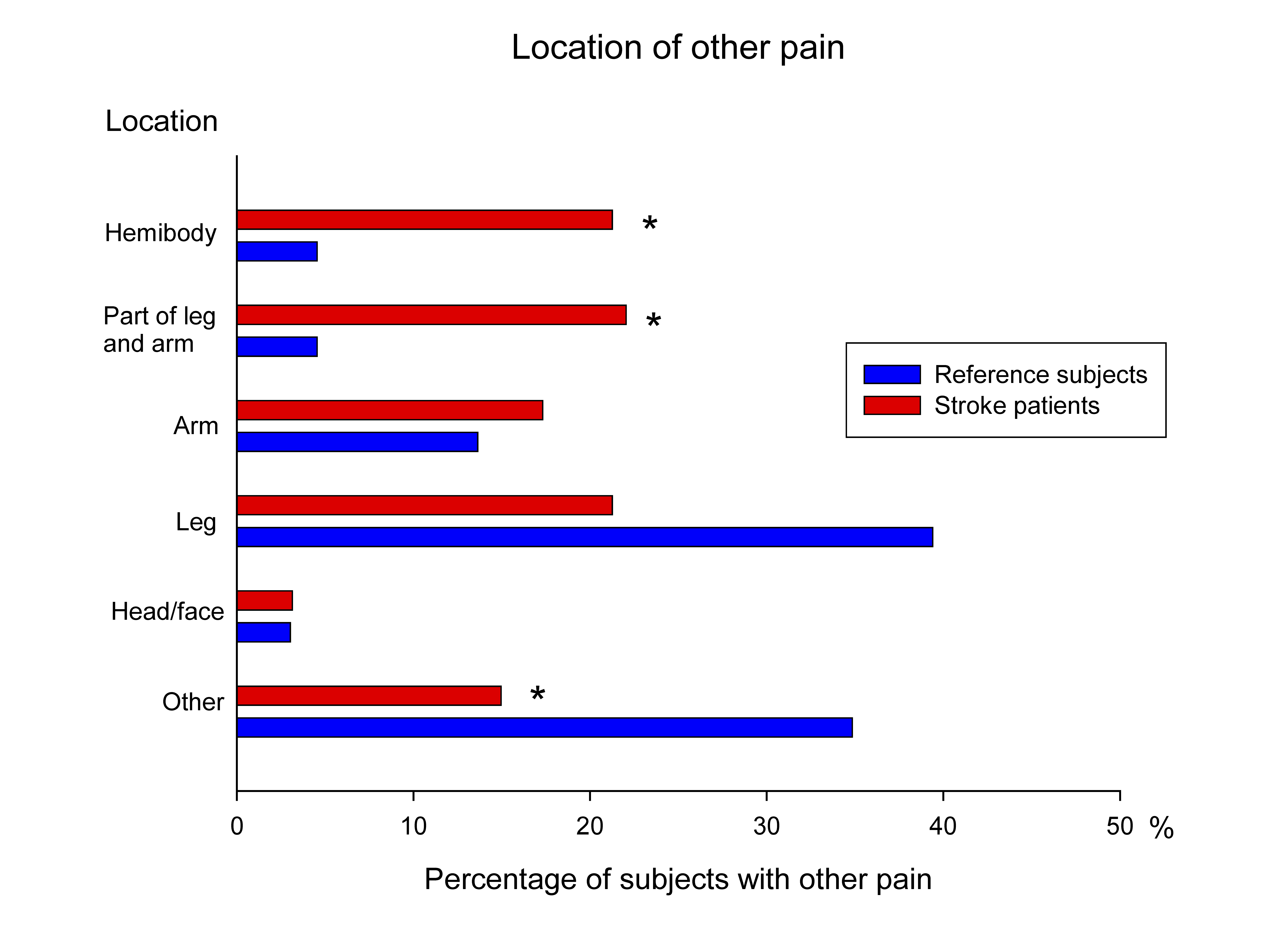

Supplement: Figure S3 — Localization of other novel pain. The reported location of worst “other pain” in stroke patients (red bars) and reference subjects (blue bars). A hemibody distribution of pain, i.e., pain localized to one side of the body, with or without involvement of the face and trunk, and pain in parts of both upper and lower limbs, was more common in stroke patients than in reference subjects (hemibody: 21.3% vs. 4.5%, p = 0.002; parts of upper and lower limbs: 22.1% vs. 4.6%, p = 0.002), whereas pain with other localizations, including wide spread pain, pain in multiple sites, back pain and neck pain, was more common in reference subjects (15.0% vs. 34.9%, p = 0.003). (TIF) [file pone.0027607.s003.tif]
