# Supplementary material for: Pain Following Stroke: A Population-Based Follow-Up Study
Source: PLoS One. 2011 Nov 15;6(11):e27607. doi: 10.1371/journal.pone.0027607 (PMC3216963; doi:10.1371/journal.pone.0027607)
Supplement: Table S1 — Characterization of the included stroke patients at time of stroke based on NIP data. (DOC) [file pone.0027607.s004.doc]

| **Risk factor** | **Included stroke patients**  **% (n/N)** |
| --- | --- |
| Number of stroke patients | 608 |
| Age at time of stroke (years), median (p10-90) | 69.3 (50.5-82.7) |
| Male percentage (%) | 55.9 |
| Ischemic stroke, n (%) | 70.5% (429/608) |
| [Hemorrhagic](http://en.wikipedia.org/wiki/Hemorrhage) stroke, n (%) | 12.2% (74/608) |
| Unspecified stroke, n (%) | 17.3% (105/608) |
| SSS score at admission, median (p10-90)* | 50 (26-58) |
| SSS score ≥ 45 (%) | 67.2% (354/527) |
| Barthel Index score (post-stroke day 7), median (p10-90)# | 90 (15-100) |
| Barthel Index score ≥80 (%) | 61.1% (289/473) |
| Diagnosed with diabetes at the time of stroke (%) | 13.5% (80/593) |
| Diagnosed with atria fibrillation at the time of stroke (%) | 12.0% (71/593) |
| History of MI at time of stroke (%) | 10.7% (64/596) |
| Diagnosed with hypertension at time of stroke (%) | 56.1% (341/608) |
| History of previous stroke (%) | 23.1% (138/597) |
| Intermittent claudication at time of stroke (%) | 5.6% (32/569) |
| Diagnosed with hyperlipidemia at time of stroke (%) | 49.7% (280/563) |
| Diagnosed with carotid stenosis at time of stroke (%) | 10.9% (31/285) |
|  |  |

**eTable 1: Characterization of included stroke patients at time of stroke**
