# Supplementary material for: Pain Following Stroke: A Population-Based Follow-Up Study
Source: PLoS One. 2011 Nov 15;6(11):e27607. doi: 10.1371/journal.pone.0027607 (PMC3216963; doi:10.1371/journal.pone.0027607)
Supplement: Table S2 — Characterization of stroke patients and reference subjects with development of all types of novel pain and subtypes of pain. (DOC) [file pone.0027607.s005.doc]

|  |  | **Stroke**  **patients** | **Reference subjects** | **P-value** | **Odds ratio** |
| --- | --- | --- | --- | --- | --- |
| **Chronic pain (all types) (n)** | | 39.0% (237/608) | 28.9% (150/519) | **<0.001** | 1.57 (1.21-2.04) |
|  | Median age (years) (p10-p90) | 70.8 (51.9-85.2) | 74.0 (56.5-85.5) | 0.077 |  |
|  | Gender (male %) | 52.3% | 48.7% | 0.48 |  |
|  | Age < 65 years (n) | 46.2% (86/186) | 23.8% (41/172) | **<0.001** | 2.75 (1.71-4.45) |
|  | Age 65-74 years (n) | 33.7% (58/172) | 26.4% (39/148) | 0.15 | 1.42 (0.85-2.38) |
|  | Age ≥ 75 years (n) | 37.2% (93/250) | 35.2% (70/199) | 0.66 | 1.09 (0.73-1.64) |
| **Headache (n)** | | 10.5% (64/608) | 2.3% (12/519) | **<0.001** | 4.97 (2.62-10.23) |
|  | Median age (years) (p10-p90) | 66.1 (48.8-82.5) | 57.5 (49.0-82.0) | 0.37 |  |
|  | Gender (male %) | 50.0% | 66.7% | 0.29 |  |
|  | Age < 65 years (n) | 15.1% (28/186) | 4.7% (8/172) | **0.001** |  |
|  | Age 65-74 years (n) | 10.5% (18/172) | 0% (0/148) | **<0.001** |  |
|  | Age ≥ 75 years (n) | 7.2% (18/250) | 2.0% (4/199) | **0.014** |  |
|  | More than 7 days/month | 6.6% (40/608) | 1.2% (6/519) | **<0.001** | 6.02 (2.51-17.50) |
|  | Severe or unbearable | 4.9% (30/608) | 1.0% (5/519) | **<0.001** | 5.34 (2.03-17.72) |
|  | History of prior headache | 46.9% (30/64) | 91.7% (11/12) | **0.004** |  |
| **Shoulder pain** | | 15.1 (92/608) | 9.8 (51/519) | **0.005** | 1.64 (1.12-2.40) |
|  | Median age (years) (p10-p90) | 66.2 (48.8-82.4) | 72.0 (57.0-87.0) | **0.028** |  |
|  | Gender (male %) | 53.3% | 47.1% | 0.30 |  |
|  | Age < 65 years | 22.6% (42/186) | 9.3% (16/172) | **0.001** | 2.84 (1.48-5.65) |
|  | Age 65-74 years | 13.4% (23/172) | 9.5% (14/148) | 0.28 | 1.48 (0.70-3.24) |
|  | Age ≥ 75 years | 10.8% (27/250) | 10.6% (21/199) | 0.93 | 1.03 (0.54-1.98) |
| **Other joint pain** | | 22.0% (134/608) | 18.5% (96/519) | 0.14 | 1.25 (0.92-1.69) |
|  | Median age (years) (p10-p90) | 66.8 (49.7-82.5) | 71.6 (55-86) | **0.0081** |  |
|  | Gender (male %) | 47.8% | 47.9% | 0.98 |  |
| **Muscle stiffness and spasms** | | 32.8% (192/586) | 9.8% (49/501) | **<0.001** | 4.50 (3.16-6.46) |
|  | Pain due to muscle stiffness and spasms | 17.4% (102/586) | 5.2% (26/501) | **<0.001** | 3.85 (2.43-6.28) |
|  | Median age (years, p10-p90) | 68.4 (51.7-85.1) | 72.5 (57.0-85.0) | 0.17 |  |
|  | Gender (male %) | 55.9% | 42.3% | 0.22 |  |
| **Other novel pain (n)** | | 22.9% (139/608) | 13.5% (70/519) | **<0.001** | 1.90 (1.37-2.64) |
|  | Median age (years) (p10-p90) | 69.4 (50.8-85.3) | 73.5 (57.0-85.0) | 0.16 |  |
|  | Gender (male %) | 48.9% | 55.7% | 0.35 |  |
|  | Age < 65 years (n) | 28.5% (53/186) | 11.6% (20/172) | **<0.001** | 3.03 (1.67-5.62) |
|  | Age 65-74 years (n) | 21.5% (37/172) | 12.8% (19/148) | **0.042** | 1.86 (0.98-3.61) |
|  | Age ≥ 75 years (n) | 19.6% (49/250) | 15.6% (31/199) | 0.27 | 1.32 (0.79-2.25) |
|  | Unilateral pain distribution (%) | 71.2% | 32.9% | **<0.001** |  |
|  | Bilateral or midline pain distribution (%) | 24.5% | 65.7% | **<0.001** |  |
|  | Hemibody pain (with or without face) (%) | 20.9% | 4.3% | **0.002** |  |
|  | Involvement of both upper and lower limb (%) | 20.1% | 4.3% | **0.002** |  |
|  | Involvement of one limb (%) | 40.3% | 54.3% | 0.055 |  |
|  | Other localizations (e.g., multiple sites, back, neck, face) (%) | 15.8% | 35.7% | **0.001** |  |
|  | Median NRS (0-10) | 5 (3-8) | 5 (2-8) | 0.14 |  |
|  | “Pins and needles” | 59.0% (82/139) | 40.0% (28/70) | **0.009** |  |
|  | Dysesthesia or allodynia to touch | 64.8% (90/139) | 18.6% (13/70) | **<0.001** |  |
|  | Burning pain | 19.4% | 14.3% | 0.36 |  |

Table e-1. Characterization of stroke patients and reference subjects with development of novel pain in general and subtypes of pain.
